# MiR-1231 decrease the risk of cancer-related mortality in patients combined with non-small cell lung cancer and diabetes mellitus

**DOI:** 10.1186/s12935-020-01525-z

**Published:** 2020-09-07

**Authors:** Jing Li, Jialiang Xu, Zhijun Cao, Shouzuo Du, Luyu Zhang

**Affiliations:** 1 Department of Endocrinology, Suzhou Xiangcheng People’s Hospital, Suzhou, China; 2 Department of Medicine, Respiratory, Emergency and Intensive Care Medicine, The Affiliated Dushu Lake Hospital of Soochow University, Suzhou, China; 3 Department of Cardiovascular, The First Affiliated Hospital of Soochow University, Suzhou, China; 4 Department of Urology, The Ninth People’s Hospital of Suzhou, Suzhou, China

**Keywords:** Non-small cell lung cancer, Diabetes mellitus, miR-1231, Overall survival, The prediction model

## Abstract

**Background:**

Non-small cell lung cancer (NSCLC) is a deadly human malignancy, and previous studies support the contribution of microRNAs (miRNAs) to cancer assessment. It has been reported that miR-1231 can be used as a biomarker to assess prognosis in different cancers. However, the prognostic value of miR-1231 in NSCLC patients with comorbid diabetes mellitus (DM) remains unclear. The present study evaluated the risk factors for NSCLC with DM and developed a predictive model for it.

**Methods:**

A real-world study was conducted, including data from 108 patients with NSCLC combined with DM from April 1, 2010, to June 1, 2015. MiR-1231 was recorded during hospital admission. Cox-proportional hazards model was applied for survival analysis of risk factors for cancer-related mortality and to create nomograms for prediction. The accuracy of the model was evaluated by C-index and calibration curves.

**Results:**

The mortality rate in the high miR-1231 level (≥ 1.775) group was 57.4%. On the basis of univariate analysis, we put factors (P < 0.05) into multivariate regression models, and high miR-1231 levels (P < 0.001, HR = 0.57), surgery (P < 0.001, HR = 0.37) and KPS score > 80 (P = 0.01, HR = 0.47) had a better prognosis and were considered as independent protective factors. These independently relevant factors were used to create nomograms to predict long-term patient survival. Nomogram showed good accuracy in risk estimation with a guide-corrected C-index of 0.691.

**Conclusion:**

MiR-1231 reduced the risk of cancer-related death in patients with combined NSCLC and DM. Nomogram based on multivariate analysis showed good accuracy in estimating the overall risk of death.

## Background

Lung cancer is the most common cancer and is the leading cause of cancer-related deaths worldwide [1]. Lung cancer is divided by pathology into small cell lung cancer and non-small cell lung cancer (NSCLC), which account for 15% and 85% of lung cancer cases, respectively [2]. Currently, the standard treatment for NSCLC includes surgery, chemotherapy, radiation therapy, and targeted therapy [3]. Despite recent advances in diagnostic and therapeutic strategies, the 5-year survival rate for NSCLC is still less than 20% [4]. Therefore, the search for more effective indicators for early diagnosis and prognosis prediction has become urgent.

MicroRNAs (miRNAs) are a group of highly conserved non-coding RNAs (ncRNAs) that are 20-24 nucleotides in length [5]. In general, miRNAs can inhibit mRNA translation or promote RNA degradation by binding to the 3′- UTRs of target genes [6]. Many evidence suggests that aberrant expression and regulation of miRNAs play an essential role in the development of human cancers [7]. Some miRNAs can affect many types of malignancies and serve as biomarkers for these cancers [8]. To date, several miRNAs have been shown to play an essential role in the development of NSCLC, but few are likely to be biomarkers or candidates for targeted therapies.

Recently, miR-1231 is dysregulated and plays a vital role in different cancers. miR-1231 was found to be significantly decreased in prostate cancer (PC) tissues compared to healthy tissues and could be a prognostic biomarker and therapeutic target in PC patients [9]. The study by Zhang et al. revealed that miR-1231 inhibits tumor suppression in glioma by targeting the EGFR role [10]. Wang et al. showed that low expression of miR-1231 had a significant impact on overall survival (OS) and progression-free survival (PFS) in glioma and demonstrated that it might be an independent prognostic factor for glioma patients [11]. However, the biological function of miR-1231 and its potential prognostic role as a biomarker in NSCLC is unclear.

Diabetes mellitus (DM) is a growing epidemic influenced by genetic and environmental factors worldwide [12]. The development of hyperglycemia can lead to the development of several diseases. In general, the complications of DM increase the mortality of patients [13]. Many studies have shown a significant link between aberrant miRNA expression and DM [14, 15].

In this study, we aimed to detect changes in miR-1231 expression levels in NSCLC patients with DM and to explore the potential prognostic role of miR-1231. We also demonstrate a nomogram that can provide individualized, evidence-based, and highly accurate risk estimates. Nomograms are easy to use and can facilitate management-related decisions.

## Methods

### Study design and participant characteristics

**Fig. 1:**
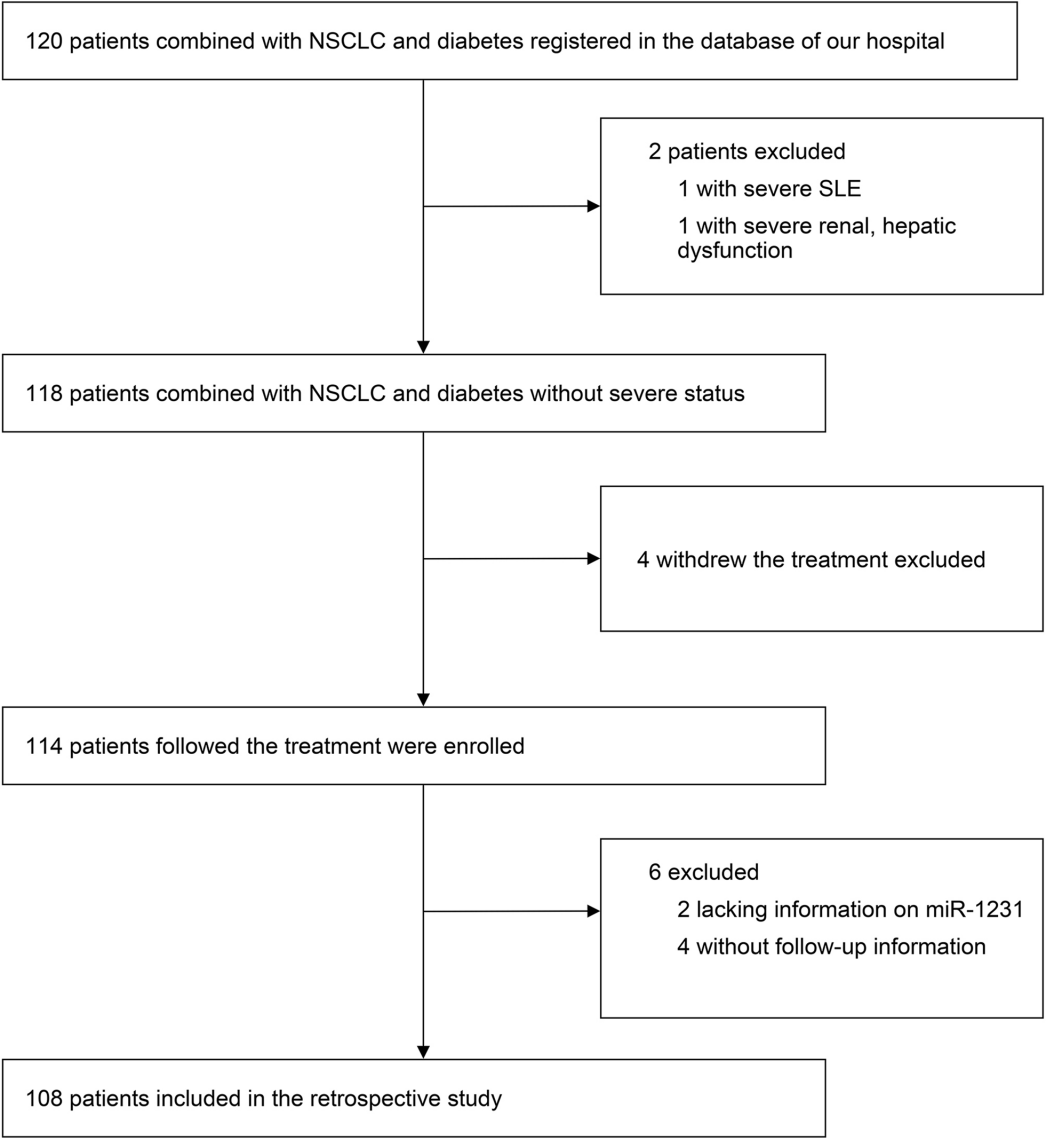
A flow chart of the screening process

We did a real-world study, including data from 108 patients with NSCLC and DM from April 2010 to June 2015 at Suzhou Xiangcheng People’s Hospital, Suzhou, China. Those who lacked miRNA data information withdrew from treatment or had no follow-up information were excluded. The flowchart of the screening process is shown in Fig. 1. Patient sex, age, BMI, serum CEA level, albumin level, CRP level, hemoglobin level, neutrophil count, lymphocyte count, platelet count, NSCLC stage, PNI score, KPS score, NLR, pathology type, surgery, radiotherapy, application of platinum, targeted therapy, application of VEGF inhibitors, application of TKI, smoking, diabetes mellitus, ACS, hyperlipidemia, and heart failure were recorded. Patients were diagnosed with NSCLC by histopathological examination. The definition of DM and details of all the above variables are given in Part I of Additional file 1. The median follow-up period was 20.8 months. Informed, and consent was obtained from all patients or their immediate family members. All protocols were following the guidelines of the ethics committee of Suzhou Xiangcheng People’s Hospital and followed the Declaration of Helsinki.

### Assays for detection of miR-1231 levels

The quantitative reverse transcription real-time polymerase chain reaction (qRT-PCR) was performed to detect miR-1231 expression levels.

Total RNA from tissues was isolated and extracted by miRcute Extraction and Separation of miRNAs kit (Tiangen Biotech Co., Ltd., Beijing, China), and then reversely transcribed into cDNA using PrimeScript™ 1st strand cDNA synthesis kit (Takara Biotechnology Co., Ltd., Dalian, China) according to the manufacturer’s protocol. SYBR PrimeScript miRNA RT-PCR kit (Takara Biotechnology Co., Ltd.) was applied for qRT-PCR. The thermocycling conditions were as follows: one cycle at 95 °C for 3 min (initial denaturation), 40 cycles at 95 °C for 15 s, and 60 °C for 30 s. U6 small nuclear RNA (U6) was used as internal control. The relative expression of miR-1231 was quantified using the 2^−ΔΔCt^ methods and normalized to U6. The experiments were repeated at least three times. The following primers were used: miR-1231 forward, 5′-GCCAGTGTCTGGGCGGAC-3′ and reverse, 5′-GTGCAGGGTCCGAGGT-3′; U6 forward, 5′-CTGGTTAGTACTTGGACGGGAGAC-3′ and reverse, 5′-GTG CAGGGTCCGAGGT-3′.

### Statistical analysis

The sample size was assessed using NCSS-PASS software version 11.0 (https://www.ncss.com/software/pass/). The power was set to 0.99 and α to 0.5. The mortality rates (0.750 and 0.950) for the miR-1231 high-level group and the miR-1231 low-level group from previous data (2008–2009) were entered into PASS. The actual hazard ratio was set to 0.45. Then the sample size was calculated by PASS with a minimum sample size of 85 (control group = 42, experimental group = 43). Our sample size of 108 (54 per group) was appropriate. The sample size assessment is reported in Part II of Additional file 1. Missing data (< 5.0%) were estimated by the random forest algorithm using the ‘mouse’ package from RStudio (R version 3.6.1). Categorical variables were expressed as percentages and compared by the κ^2^ test. Skewed and normally distributed continuous variables are presented as median versus quartile range and mean ± standard deviation. Comparisons between groups were made using the Mann–Whitney U test and unpaired *t* test. Cumulative mortality rates are presented using Kaplan–Meier curves and analyzed using the log-rank test. Univariate and multivariate survival analyses of OS were evaluated using Cox regression models. Forest plots were applied to visualize the significance of covariates on prognosis. Restricted cubic pinch line analysis was performed using Harrell’s regression modeling strategy (rms) package.

To model prognostic risk, Lasso regression was performed to identify risk factors associated with prognosis. Each covariate’s contribution was quantified and visualized in the prognostic nomogram, which was internally validated by 1000 bootstrapping. The consistency of the resulting model was assessed by calibration testing. Decision curve analysis was used to assess the model’s net clinical benefit compared to traditional prognostic scores. Scatter plots were used to visualize the concordance of each model. One thousand bootstraps were used as indicated. Kaplan–Meier curves and log-rank tests were used to assess the association between miR-1231 class and survival endpoints. Statistical analysis was performed using RStudio (R version 3.6.1) with the following package rows.’ ‘ggplot2′, ‘rms’, ‘PredictABLE’, ‘risk regression’ and ‘survminer’.

## Results

### Baseline characteristics

A total of 108 patients with NSCLC, as well as DM between April 2010 and June 2015, were included in this study. The median age was 64 years (58–70 years) and included 74 (69.0%) males. The median serum CEA and CRP levels were 3.90 ng/mL and 9.49 μmol/L, respectively. The pathological types of these patients were as follows: adenocarcinoma 58 (54.0%), mixed lung cancer 17 (16.0%), large cell lung cancer 5 (5.0%), squamous cell carcinoma 27 (25.0%), and other types 1 (1.0%). For the stage of NSCLC, 17 (16.0%) patients were diagnosed with stage I, 8 (7.0%) with stage II, 20 (19.0%) with stage III, and 63 (58.0%) with stage IV. 42 (40.0%) patients underwent surgery. 43 (43.0%) patients underwent radiation therapy. In addition, 105 (97.0%) patients used platinum-based drugs and 26 (24.0%) patients used TKI. The KPS scores of these patients were examined and showed that 94 (87.0%) patients obtained a score of 80 or higher. The distribution of underlying diseases was also assessed in our data. Cardiovascular diseases such as heart failure and ACS were present in two (2.0%) and three (3.0%) patients. Twenty (19.0%) patients had hyperlipidemia. Sixty-nine (64.0%) patients had hypertension. Besides, 57 (53.0%) patients had a smoking habit. The baseline characteristics of these patients are listed in Table 1.

**Table 1:** Study participant characteristics at enrollment

Variation	Total (n = 108)	Cohort, median (IQR)		P value
		miR-1231 < 1.775	miR-1231 ≥ 1.775	
Age (year)	64.08 ± 7.6	64.69 ± 7.76	63.48 ± 7.47	0.413
BMI (kg/m^2^)	23.9 ± 3.53	23.82 ± 3.75	23.98 ± 3.33	0.819
Serum CEA level (ng/mL)	3.9 (1.83, 13.43)	5.92 (2.41, 38.39)	3.12 (1.47, 6.91)	0.021*
Serum CRP level (μmol/L)	9.49 (2.38, 13.34)	11 (4.26, 13.28)	5.99 (2.14, 13.37)	0.227
Albumin level (g/L)	40.01 ± 5.21	39.46 ± 4.83	40.57 ± 5.56	0.272
Neutrophils count (10^9^/L)	4.68 (3.53, 6.27)	4.72 (3.67, 6.08)	4.68 (3.37, 6.4)	0.549
Lymphocytes count (10^9^/L)	1.68 (1.23, 2.15)	1.47 (1.18, 2.02)	1.75 (1.36, 2.3)	0.073
Hemoglobin level (g/L)	133 (123, 142.25)	133.5 (123.25, 144.75)	131.5 (123, 141.75)	0.412
Platelet count (10^9^/L)	228.5 (186, 289)	225.5 (183.25, 291)	243.5 (200.25, 283.25)	0.365
PNI score	49.22 (44.03, 53.94)	47.62 (43.12, 51.64)	50.05 (44.48, 55.7)	0.101
NLR	2.9 (2.01, 4.49)	3.19 (2.37, 4.66)	2.69 (1.86, 3.87)	0.098
Gender (n %)				0.534
Female	34 (31)	15 (28)	19 (35)	
Male	74 (69)	39 (72)	35 (65)	
Pathologic type (n %)				0.547
Adenocarcinoma	58 (54)	29 (54)	29 (54)	
Mixed lung cancer	17 (16)	7 (13)	10 (19)	
Large cell lung cancer	5 (5)	4 (7)	1 (2)	
Squamous carcinoma	27 (25)	14 (26)	13 (24)	
Others	1 (1)	0 (0)	1 (2)	
Metastasis, n (%)				0.177
No	50 (46)	21 (39)	29 (54)	
Yes	58 (54)	33 (61)	25 (46)	
Stage of NSCLC				0.005**
Stage I	17 (16)	4 (7)	13 (24)	
Stage II	8 (7)	6 (11)	2 (4)	
Stage III	20 (19)	6 (11)	14 (26)	
Stage IV	63 (58)	38 (70)	25 (46)	
Surgery (n %)				0.001**
No	66 (61)	42 (78)	24 (44)	
Yes	42 (39)	12 (22)	30 (56)	
Therapy of radiation (n %)				0.238
No	65 (60)	29 (54)	36 (67)	
Yes	43 (40)	25 (46)	18 (33)	
Application of platinum (n %)				1
No	3 (3)	2 (4)	1 (2)	
Yes	105 (97)	52 (96)	53 (98)	
Chemotherapy				0.573
AP	45 (42)	25 (46)	20 (37)	
DP	13 (12)	6 (11)	7 (13)	
EP	14 (13)	8 (15)	6 (11)	
GP	8 (7)	2 (4)	6 (11)	
Others	28 (26)	13 (24)	15 (28)	
Target therapy (n %)				0.02*
No	76 (70)	32 (59)	44 (81)	
Yes	32 (30)	22 (41)	10 (19)	
Application of TKI (n %)				0.006**
No	82 (76)	34 (63)	48 (89)	
TKI I	19 (18)	15 (28)	4 (7)	0.006**
TKI III	7 (6)	5 (9)	2 (4)	
Application of VEGF inhibitor, n (%)				0.775
No	94 (87)	48 (89)	46 (85)	
Yes	14 (13)	6 (11)	8 (15)	
KPS score, n (%)				0.013*
20	1 (1)	0 (0)	1 (2)	
50	3 (3)	2 (4)	1 (2)	
60	1 (1)	1 (2)	0 (0)	
70	9 (8)	6 (11)	3 (6)	
80	31 (29)	22 (41)	9 (17)	
90	40 (37)	16 (30)	24 (44)	
100	23 (21)	7 (13)	16 (30)	
Smoking, n (%)				1
No	51 (47)	26 (48)	25 (46)	
Yes	57 (53)	28 (52)	29 (54)	
Hypertension, n (%)				1
No	39 (36)	20 (37)	19 (35)	
Yes	69 (64)	34 (63)	35 (65)	
Hyperlipemia, n (%)				0.215
No	88 (81)	41 (76)	47 (87)	
Yes	20 (19)	13 (24)	7 (13)	
Heart failure, n (%)				0.495
No	106 (98)	52 (96)	54 (100)	
Yes	2 (2)	2 (4)	0 (0)	
ACS, n (%)				1
No	105 (97)	52 (96)	53 (98)	
Yes	3 (3)	2 (4)	1 (2)	

IQR, interquartile range; CRP, C-reactive protein; PNI, neutrophil lymphocyte ratio; NLR, neutrophil lymphocyte ratio; NSCLC, non-small-cell lung cancer; TKI, Tyrosine Kinase Inhibitor; VEGF, vascular endothelial growth factor; KPS, Karnofsky Performance Status; ACS, acute coronary syndrome. ***P < 0.001, **P < 0.01, *P < 0.05

The overall mortality rate was 78.7% in all 108 patients. The mortality rate in the high miR-1231 level group was 57.4%. Also, in the high miR-1231 level group, there were 25 patients (46.0%) in stage IV compared to 38 patients (70.0%) in the low group (Table 1).

### MiR-1231 expression level and clinical risk factors predict the development of NSCLC patients with DM

**Fig. 2:**
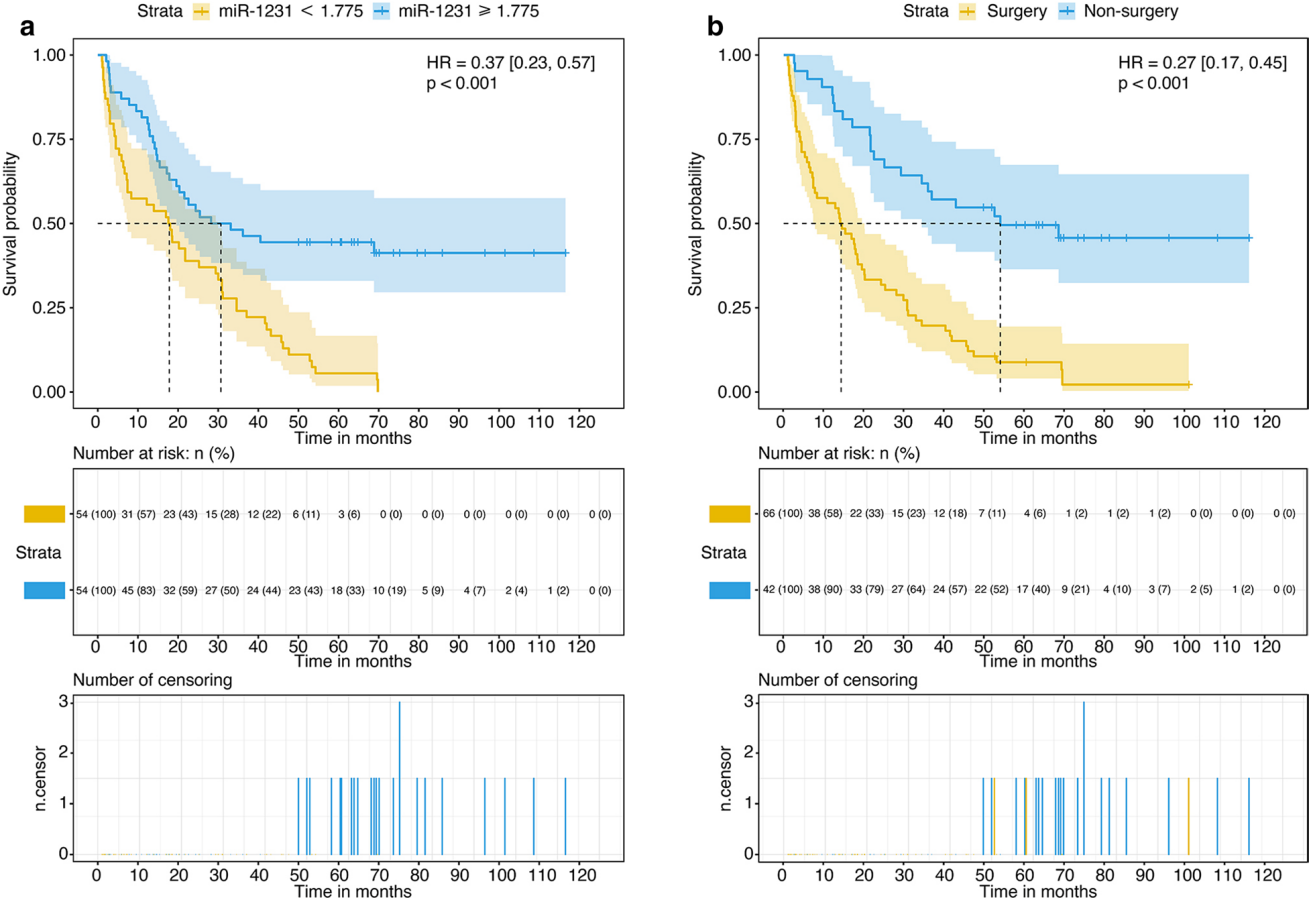
Overall survival (OS) of NSCLC patients with diabetes mellitus in different levels of miR-1231 and different treatments. **a** OS of patients with a high or low level of miR-1231. **b** OS of patients with different treatments (surgery vs. non-surgery)

Based on univariate analysis, high miR-1231 levels (≥ 1.775) were a robust protective predictor of cancer-related mortality (HR 0.37, 95% CI 0.23-0.57, P < 0.001) (Table 2). Kaplan–Meier curves showed that patients in the high miR-1231 group had a lower cumulative mortality rate than those in the low miR-1231 group (log-rank P < 0.001) (Fig. 2a). Also, in the survival curve, patients who underwent surgery had lower mortality rates compared to those who did not (HR 0.27, 95% CI 0.17-0.45, P < 0.001) (Fig. 2b).

**Table 2:** Cox regression analysis of hazard ratio on NSCLC patients with diabetes mellitus

Variation	Non-adjustment		Model 1	
	Hazard ratio (95% CI)	P value	Hazard ratio (95% CI)	P value
Gender, male vs. female	1.68 [1.04, 2.73]	0.035*	–	–
Age (year), ≥ 60 vs. < 60	1.20 [0.76, 1.89]	0.443	–	–
BMI, ≥ 23.9 kg/m^2^ vs. < 23.9 kg/m^2^	0.66 [0.43, 1.02]	0.059	0.68 [0.44, 1.05]	0.08
Serum CEA level, > 3.9 ng/mL vs. ≤ 3.9 ng/mL	1.43 [0.93, 2.19]	0.106	1.35 [0.88, 2.09]	0.172
Serum CRP level, > 9.49 μmol/L vs. ≤ 9.49 μmol/L	2.18 [1.41, 3.38]	< 0.001***	2.08 [1.34, 3.23]	0.001**
Albumin level, ≤ 40.01 g/L vs. > 40.01 g/L	2.67 [1.73, 4.13]	< 0.001***	2.53 [1.55, 4.11]	< 0.001***
Neutrophils count, > 4.68 × 10^9^/L vs. ≤ 4.68 × 10^9^/L	1.77 [1.15, 2.73]	0.009**	1.61 [1.01, 2.57]	0.046***
Lymphocytes count, > 1.68 × 10^9^/L vs. ≤ 1.68 × 10^9^/L	0.70 [0.46, 1.08]	0.104	0.70 [0.46, 1.08]	0.109
Hemoglobin level, > 133 g/L vs. ≤ 133 g/L	0.70 [0.45, 1.07]	0.095	0.60 [0.39, 0.94]	0.024**
Platelet count, > 228.5 × 10^9^/L vs. ≤ 228.5 × 10^9^/L	1.42 [0.92, 2.18]	0.109	1.53 [0.99, 2.36]	0.057
PNI score, > 49.22 vs. ≤ 49.22	0.40 [0.26, 0.62]	< 0.001***	0.42 [0.27, 0.66]	< 0.001***
NLR, > 2.9 vs. ≤ 2.9	1.85 [1.20, 2.85]	0.005**	1.71 [1.09, 2.67]	0.019*
Pathologic type, Adenocarcinoma vs. others	1.01 [0.66, 1.54]	0.975	1.13 [0.72, 1.76]	0.601
Metastasis, Yes vs. No	1.66 [1.06, 2.60]	0.026*	1.70 [1.09, 2.65]	0.02*
Stage of NSCLC, IV, III vs. II and I	2.01 [1.12, 3.62]	0.019*	1.86 [1.01, 3.42]	0.048*
Surgery, Yes vs. No	0.27 [0.17, 0.45]	< 0.001***	0.26 [0.15, 0.43]	< 0.001***
Therapy of radiation, Yes vs. No	1.36 [0.88, 2.09]	0.163	1.34 [0.85, 2.11]	0.21
Application of platinum, Yes vs. No	0.27 [0.08, 0.87]	0.028*	0.32 [0.10, 1.06]	0.062
Target therapy, Yes vs. No	1.03 [0.65, 1.62]	0.903	1.09 [0.69, 1.72]	0.724
Application of TKI, Yes vs. No	1.12 [0.69, 1.81]	0.644	1.15 [0.71, 1.87]	0.567
Application of VEGF inhibitor, Yes vs. No	0.81 [0.40, 1.61]	0.539	0.74 [0.37, 1.48]	0.389
Chemotherapy, AP vs. others	0.83 [0.54, 1.28]	0.407	0.91 [0.59, 1.42]	0.685
Smoking, Yes vs. No	1.45 [0.94, 2.24]	0.095	1.02 [0.56, 1.87]	0.936
Hypertension, Yes vs. No	0.96 [0.62, 1.49]	0.854	0.88 [0.56, 1.39]	0.59
Hyperlipemia, Yes vs. No	0.61 [0.34, 1.09]	0.096	0.61 [0.34, 1.08]	0.089
Heart failure, Yes vs. No	12.63 [2.84, 56.17]	0.001**	10.67 [2.38, 47.88]	0.002**
ACS, Yes vs. No	0.73 [0.18, 2.96]	0.656	0.82 [0.20, 3.37]	0.787
KPS score, > 80 vs. ≤ 80	0.27 [0.17, 0.43]	< 0.001***	0.27 [0.17, 0.43]	< 0.001***
miR-1231, > 1.78 vs. ≤ 1.78	0.37 [0.23, 0.57]	< 0.001	0.36 [0.23, 0.57]	< 0.001***

Model 1: Adjusted by age and gender

HR, hazard risk; BMI, Body Mass Index; CRP, C-reactive protein; PNI, neutrophil lymphocyte ratio; NLR, neutrophil lymphocyte ratio; NSCLC, non-small-cell lung cancer; TKI, Tyrosine Kinase Inhibitor; VEGF, vascular endothelial growth factor; KPS, Karnofsky Performance Status; ACS, acute coronary syndrome. ***P < 0.001, **P < 0.01, *P < 0.05

In addition, gender, serum CRP level, albumin level, neutrophil count, PNI score, NSCLC staging, platinum application, NLR, metastasis, surgery, heart failure, and KPS score were also associated with overall mortality (Table 2). When adjusted for age and sex, patients in the high miR-1231 level group also had a lower incidence of cumulative mortality than those in the low-level group.

### Independent prognostic factors for OS of NSCLC patients with DM

**Fig. 3:**
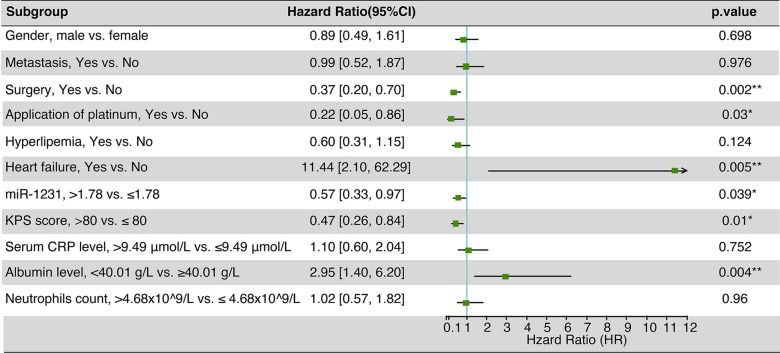
Multivariate cox regression analysis of 5-year overall survival on data in the NSCLC patients with diabetes mellitus

After multivariate adjustment, high miR-1231 levels (HR 0.57, 95% CI 0.33–0.97, P < 0.001) were also associated with a low increased risk of death (Fig. 3). Also, surgery, application of platinum, albumin level, and heart failure were independent risk factors.

### Development and validation of an OS-predicting nomogram

**Fig. 4:**
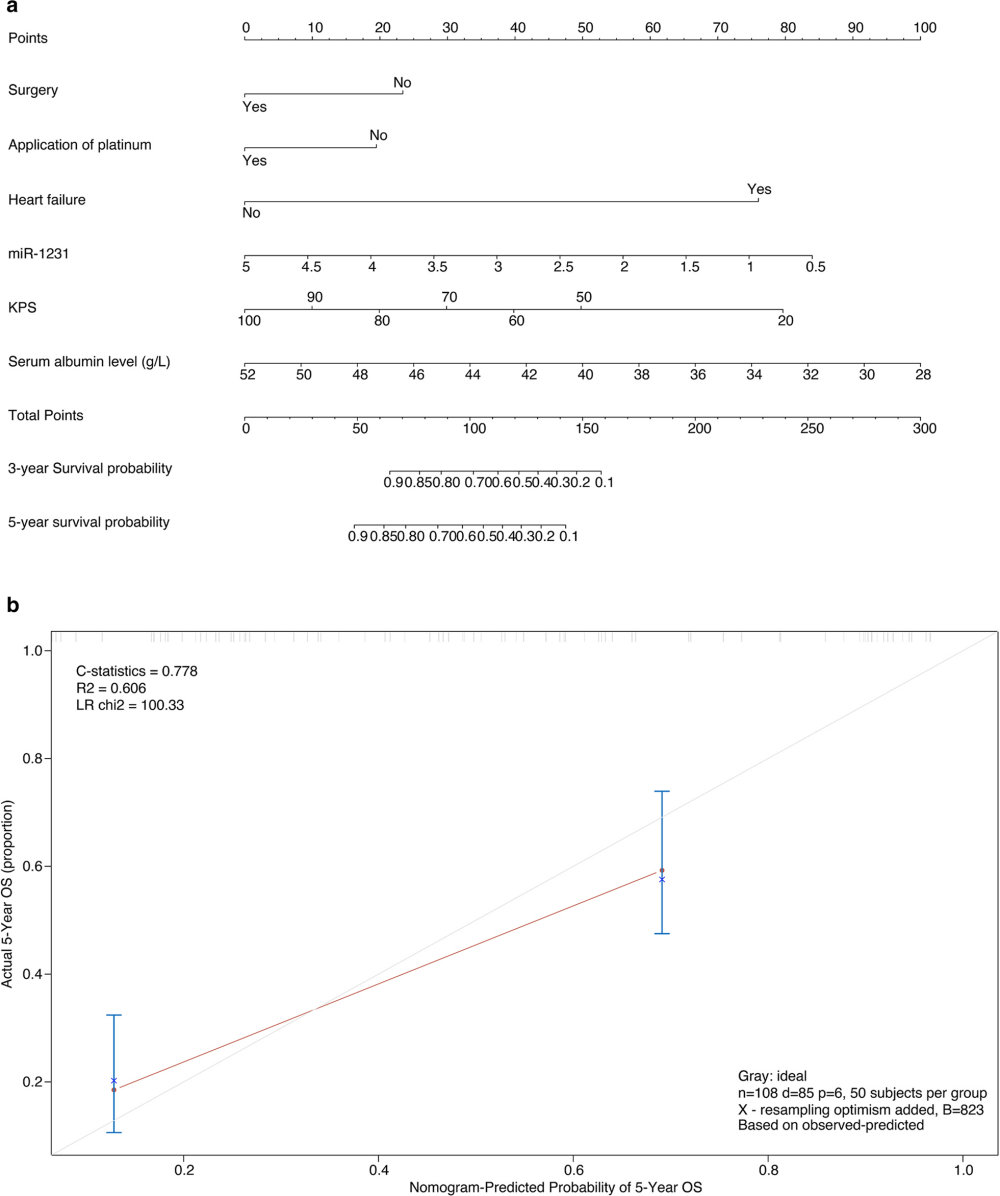
Nomogram for overall survival (OS) risk estimation of NSCLC patients with diabetes mellitus and its predictive performance. **a** Nomogram to estimate the OS risk of patients in different variations. To build the nomogram, find the position of each variable on the corresponding axis, draw a line to the points axis for the number of points, add the points from all of the variables, and draw a line from the total points axis to determine the OS probabilities at the lower line of the nomogram. **b** The validity of the predictive performance of the nomogram in estimating the OS risk of NSCLC patients with diabetes mellitus

The independent risk factors derived from the multivariate analysis were used to create the OS estimation nomogram (Fig. 4a). Internal validation of the resulting model was performed by bootstrap validation. With an unadjusted C-index of 0.778 and a bootstrap correction of 0.778, the nomogram exhibited excellent accuracy in estimating OS’s risk. In the validation cohort, the nomogram has a C-index of 0.778 in estimating OS. besides, a calibration curve suitable for risk estimation (R^2^ = 0.606, LR chi2 = 100.33) is shown (Fig. 4b).

## Discussion

In this study, we examined the expression levels of miR-1231 in a large cohort of patients with NSCLC and DM at a single institution between April 2010 and June 2015. In our analysis, results showed that decreased miR-1231 expression was significantly associated with unfavorable clinical parameters. In addition, patients in the miR-1231 high expression group had a better OS compared to the miR-1231 low expression group. Multivariate analysis also showed that miR-1231 was an independent risk factor for favorable OS. In addition, these independent prognostic factors were used to form a nomogram for the estimation of OS. The nomogram showed excellent accuracy in estimating the risk of OS.

The increased incidence of NSCLC is often associated with aging, which explains why NSCLC coincides with other age-related diseases like DM [16]. Many studies have shown that preexisting DM is associated with lower 5-year survival rates in cancer patients [17–19]. Several studies have demonstrated that diabetes is associated with poor OS in lung cancer patients [20–22]. A study investigated by Ali et al. showed that diabetes at the time of diagnosis was negatively associated with the prognostic importance of OS in NSCLC patients. However, it was rarely considered in clinical studies, partly due to the lack of validated markers. Therefore, we attempted to circumvent this limitation by searching for a microRNA for predicting the prognosis of NSCLC patients with DM.

Cancer development involves several different processes, including many vital genes/proteins. Cancer signatures represent the characteristics that a cell needs to achieve to become and maintain itself as a cancer cell [23]. These signatures guide the cellular pathways associated with cancer initiation and development. Using the expression of miRNAs to predict the clinical prognosis of cancer is more advantageous than mRNAs because miRNAs are considered to be critical post-transcriptional regulators of gene expression [24, 25]. In contrast to mRNAs, these post-transcriptional regulators are highly conserved among species [26].

Previous studies have shown that miRNAs are associated with the occurrence and development of various cancers, and many miRNAs can serve as valuable biomarkers for cancer diagnosis and prognosis [8, 27]. Recently, many miRNAs have been identified as prognostic biomarkers for NSCLC [28]. miR-1298 was found by Du et al. to be aberrantly expressed in NSCLC, and aberrant miR-1298 can be used as a prognostic biomarker for NSCLS patients [29]. As a novel biomarker, miR-1231 has been considered as a promising prognostic factor for cancer. miR-1231 is a prognostic biomarker and therapeutic target in prostate cancer by Wang et al. [9]. However, no studies are examining the role of miR-1231 as a biomarker in NSCLS. To our knowledge, the present analysis is the first-ever attempt to comprehensively explore prognostic biomarkers based on miR-1231 expression in NSCLC and DM patients. In the present study, we initially tested the expression levels of miR-1231 in NSCLC and DM patients. In addition, we revealed, for the first time, the association between altered miR-1231 expression and existing clinicopathological variables. The results showed that miR-1231 was significantly associated with serum CEA levels, NSCLC stage, surgery, targeted therapy, TKI application, and KPS score. Univariate analysis showed that high expression of miR-1231 was a strong protective predictor of mortality; Kaplan–Meier curves showed that serum CEA level, NSCLC stage, surgery, targeted therapy, TKI application, and KPS score were associated with this. Patients with high miR-1231 levels or who underwent surgery had a lower cumulative incidence of death than those who underwent surgery, respectively. In addition, sex, serum CRP level, albumin level, neutrophil count, PNI score, NSCLC stage, application of platinum, NLR, metastasis, surgery, heart failure, KPS score, and miR-1231 levels were associated with overall mortality. Multivariate analysis showed that miR-1231, surgery, platinum application, albumin level, KPS score, and heart failure as independent prognostic factors predicted OS.

Nomograms help in the visualization of statistical models, calculating predictive values, and graphical assessment of the importance of variables [30]. They have been widely used to predict cancer risk and treatment outcomes. Recently, several studies have successfully created a prognostic nomogram that integrates miRNAs and clinically relevant cancer variables. However, few studies have used the combination of miR-1231 and clinical risk factors to create a prognostic model for NSCLC patients with DM. This study establishes a nomogram model capable of predicting individual prognosis in NSCLC patients with DM based on the combination of miR-1231 and independent clinicopathological characteristics. Nomogram showed good accuracy in estimating the risk of OS. The calibration curves of the risk estimates showed good agreement between observation and prediction. Thus, this is the first prognostic nomogram for patients with NSCLC and DM that considers clinical variables in addition to miR-1231. Based on this model, potentially high-risk patients can be selected for a specific treatment strategy.

There are some limitations to this study. First, experimental studies explaining the biological significance of miR-1231 are lacking. Therefore, the molecular mechanism of miR-1231 should be further investigated in NSCLC. Second, the prognostic map needs to be further validated by prospective, large-scale multicenter studies before it can be applied to clinical practice.

## Conclusions

In summary, we demonstrated that the expression pattern of miR-1231 was significantly correlated with clinical variables in NSCLC patients with DM. Furthermore, miR-1231 was shown to be an independent biomarker for predicting NSCLC patients’ prognosis with DM. Moreover, a nomogram based on multivariate analysis had good accuracy in estimating OS risk.

## Supplementary information

**Additional file 1.** Details of variants and report of sample size assessment.

## Data Availability

Not applicable.
