# Supplementary material for: MiR-1231 decrease the risk of cancer-related mortality in patients combined with non-small cell lung cancer and diabetes mellitus
Source: Cancer Cell Int. 2020 Sep 7;20:438. doi: 10.1186/s12935-020-01525-z (PMC7487554; doi:10.1186/s12935-020-01525-z)
Supplement: Supplementary file 1 — Additional file 1. Details of variants and report of sample size assessment. [file 12935_2020_1525_MOESM1_ESM.docx]

**Additional Materials**

**Part I. Details of variants**

**Patients’ age:** The age of patients was based on the time of diagnosis of NSCLC.

**Body mass index:** The equation of body mass index (BMI) is calculated as follow: BMI (kg/m^2^) = weight (kg) / height (m)^2^. The data of BMI was recorded before clinical treatment.

**Serum CEA level:** The serum level of carcinoembryonic antigen (CEA) is one of tumor marks，the higher serum *[CEA](https://www.sciencedirect.com/topics/immunology-and-microbiology/carcinoembryonic-antigen)* level than that of healthy individuals led to its clinical application as a diagnostic biomarker for cancer.

**Serum CRP level:** C-reactive protein (CRP) is detected by olorimetric determination through fasting blood collection and CRP is one of the inflamation markers.

**Albumin level:** Serum albumin is detected by colorimetric determination through fasting blood collection. And serum albumin is one of the indicators of nutritional status.

**Neutrophils count, lymphocytes count:, hemoglobin level, platelet count:** All these indexes were extracted from blood routine examination to evaluate preoperative status of patients.

**PNI score:** Prognostic nutritional index (PNI) is an index to evaluate the nutritional status of surgical patients, to predict the risk of surgery and to help make prognostic judgment. The equation is listed as below: PNI = serum albumin (g/L) + 5 * lymphocytes (*10^9^/L).

**NLR:** Neutrophil to Lymphocyte Ratio（NLR）is an index of inflammation markers to predict the prognosis of various cancer, including lung cancer.

**Pathologic type:** Non-small cell lung cancer can be classified as adenocarcinoma, squamous cell carcinoma, large cell lung cancer, or others, depending on surgical or tumor biopsy specimens

**Stage of NSCLC:** Non-small cell lung cancer was staged according to the seventh edition of international union against cancer (UICC) TNM was published in 2009

**Surgery:** For early and locally advanced NSCLC, which is generally acceptable, there is a history of surgical resection.

**Therapy of radiation:** Radiation therapy is a medical procedure that uses the delivery of high-energy radiation to kill cancer cells and shrink tumors, including external beam radiation therapy, internal radiation therapy and stereotactic body radiotherapy (SBRT). It aims to cure small tumors, treat lung cancer both locally and nearby lymph nodes, treat metastases, relieve symptoms, and also at prevention.

**Application of platinum:** Platinum-based chemotherapy may be used as a first or second line therapy for advanced lung cancer, after surgery (adjuvant chemotherapy), or before surgery to reduce the size of a tumor (neoadjuvant therapy). The most commonly used platinum drugs are cisplatin, carboplatin and nedaplatin. Treatment of [non-small cell lung cancer](https://www.verywellhealth.com/non-small-cell-lung-cancer-2249281) begins with either cisplatin or carboplatin combined with another medication, such as docetaxel, pemetrexed, paclitaxel, gemcitabine and gemcitabine.

**Target therapy:** Targeted therapies for lung cancer are drugs that are tailored to attack certain mutations of cancer cells. These mutations lead to the production of abnormal proteins which guide the growth and development of a cancer cell. Common driver mutations include EGFR mutations, ALK rearrangements, ROS1 rearrangements, MET amplifications, KRAS mutations, HER2 mutations, BRAF mutations and so on.

**Application of TKI:** Tyrosine kinase inhibitors (TKI) act as competitive inhibitors of ATP binding to tyrosine kinases, or as tyrosine analogs that block tyrosine kinase activity and inhibit cell proliferation. Epithelial growth factor receptor (EGFR) is a protein that is present on the surface of both normal cells and lung cancer cells. Mutations in EGFR can occur at different locations on exon 18 to 21. ALK-positive lung cancer refers to people who have lung cancer that tests positive for the EML4-ALK fusion mutation. Several FDA-approved medications available to treat EGFR-positive and ALK-positive lung adenocarcinoma, including:

- EFGR inhibitors - Tarceva (erlotinib), Gilotrif (afatinib), Iressa (gefitinib), Tagrisso (osimertinib), and Portrazza (necitumumab)
- ALK inhibitors - Xalkori (crizotinib), Zykadia (ceritinib), and Alecensa (alectinib)
- ROS1 inhibitor - Xalkori (crizotinib)

**Application of VEGF inhibitor:** VEGF (Vascular endothelial growth factor) can activate angiogenesis through different signaling pathways. VEGF inhibitors are drugs that block the ability of tumors to form new blood vessels, and hence, grow and spread. Some currently available medications include bevacizumab, ramucirumab, axitinib, endostar and anlotinib.

**KPS score:** A performance measure for rating the ability of a person to perform usual activities, evaluating a patient's progress after a therapeutic procedure, and determining a patient's suitability for therapy. It is used most commonly in the prognosis of cancer therapy, usually after chemotherapy and customarily administered before and after therapy. It was named for Dr. David A. Karnofsky, an American specialist in cancer chemotherapy. Patients with more than 80 scores had better postoperative status and longer survival time. And patients with more than 70 scores can suffer from chemoradiotherapy.

**Smoking:** Smoker refers to continuous or cumulative smoking > 1 cigarette/day over a lifetime of more than 6 months. (1997, WHO)

**Hypertension:** Hypertension is defined as a repeatedly elevated blood pressure exceeding 140 over 90 mmHg.

**Diabetes:** Diabetes is a group of metabolic diseases characterized by hyperglycemia. And fasting blood glucose more than 7.0 mmol/l or blood glucose more than 11.1 mmol/l within 2hoursafter meal can be diagnosed diabetes.

**Hyperlipemia:** Hyperlipemia means the presence of excess fat or lipids in the blood. And total cholesterol ≥ 6.2 mmol/L, low density lipoprotein cholesterin ≥ 4.1 mmol/L, triglyceride ≥ 2.3 mmol/L, high density lipoprotein cholesterin < 1.0 mmol/L can be diagnosed hyperlipemia.

**Heart failure:** The information was recorded through history taking and verified after hospitalization. Heart failure means inability of the heart to keep up with the demands on it and, specifically, failure of the heart to pump blood with normal efficiency. Heart failure may be due to failure of the right or left or both ventricles. The signs and symptoms depend upon which side of the heart is failing. They can include shortness of breath (dyspnea), asthma due to the heart (cardiac asthma), pooling of blood (stasis) in the general body (systemic) circulation or in the liver's (portal) circulation, swelling (edema), blueness or duskiness (cyanosis), and enlargement (hypertrophy) of the heart.

**ACS:** Acute coronary syndrome is a term for a group of conditions that suddenly stop or severely reduce blood from flowing to the heart. When blood cannot flow to the heart, the heart muscle can become damaged. Heart attack and unstable angina are both acute coronary syndromes (ACS).

**Withdraw treatment:** Reasons for patients withdrew from treatment were listed below: 1) High cost of the treatment: In China, the cost of hormone therapy can be up to 3000 RMB a month if without health insurance. 2) Poor patient compliance: Some patients discontinue treatment because they do not comply with the treatment plan prescribed by their doctor.

**Part II. Report of sample size assessment**

PASS 15.0.5 1

**Tests for Two Survival Curves Using Cox's Proportional Hazards Model**

**Numeric Results with Ha: HR ≠ 1**

**Total Control Trtmnt Prop'n Hazard Control Trtmnt**

**Sample Sample Sample Control Ratio Prob Prob Control Trtmnt**

**Size Size Size N1/N h2/h1 Event Event Events Events**

**Power N N1 N2 P1 HR Pev1 Pev2 E1 E2 Alpha Beta**

0.9005 85 42 43 0.500 0.450 0.600 0.950 25.2 40.9 0.050 0.100

**References**

Chow, S.C., Shao, J., Wang, H. 2008. Sample Size Calculations in Clinical Research, 2nd Edition. Chapman &

Hall/CRC.

Schoenfeld, David A. 1983. 'Sample Size Formula for the Proportional-Hazards Regression Model', Biometrics,

Volume 39, Pages 499-503.

**Report Definitions**

Power is the probability of rejecting a false null hypothesis. Power should be close to one.

N is the total sample size.

N1 and N2 are the sample sizes of the control and treatment groups.

P1 is the proportion of the total sample that is in the control group, group 1.

HR is the hazard ratio: h2/h1.

Pev1 and Pev2 are the probabilities of an event in the control and the treatment groups.

E1 and E2 are the number of events required in the control and the treatment groups.

Alpha is the probability of a type one error: rejecting a true null hypothesis.

Beta is the probability of a type two error: failing to reject a false null hypothesis.

**Summary Statements**

A two-sided test of whether the hazard ratio is one with an overall sample size of 85 subjects

(of which 42 are in the control group and 43 are in the treatment group) achieves 90% power at

a 0.050 significance level when the hazard ratio is actually 0.450. The number of events

required to achieve this power is 66.1. It is anticipated that proportions of subjects having

the event during the study is 0.600 for the control group and 0.950 for the treatment group.

These results assume that the hazard ratio is constant throughout the study and that Cox

proportional hazards regression is used to analyze the data.

**Procedure Input Settings**

**Autosaved Template File**

\\Mac\Home\Documents\PASS 15\Procedure Templates\Autosave\Tests for Two Survival Curves Using Cox's Proportional Hazards Model - Autosaved 2020_1_24-11_17_21.t92

**Design Tab**

Solve For: Sample Size

Alternative Hypothesis: Ha: HR ≠ 1

Power: 0.90

Alpha: 0.05

Group Allocation: Equal (N1 = N2)

Pev1 (Probability of a Control Event): 0.600

Pev2 (Probability of a Treatment Event): 0.950

HR (Actual Hazard Ratio = h2/h1): 0.45
